# Genome-centric investigation of bile acid metabolizing microbiota of dairy cows and associated diet-induced functional implications

**DOI:** 10.1038/s41396-022-01333-5

**Published:** 2022-10-19

**Authors:** Limei Lin, Zheng Lai, Huisheng Yang, Jiyou Zhang, Weibiao Qi, Fei Xie, Shengyong Mao

**Affiliations:** 1 Ruminant Nutrition and Feed Engineering Technology Research Center, College of Animal Science and Technology, Nanjing Agricultural University, Nanjing, 210095 China; 2 Laboratory of Gastrointestinal Microbiology, Jiangsu Key Laboratory of Gastrointestinal Nutrition and Animal Health, National Center for International Research on Animal Gut Nutrition, College of Animal Science and Technology, Nanjing Agricultural University, Nanjing, 210095 China

**Keywords:** Bacterial genetics, Animal physiology, Applied microbiology

## Abstract

Although the importance of bile acid (BA)-related microbial strains and enzymes is increasingly recognized for monogastric animals, a lack of knowledge about BA metabolism in dairy cows limits functional applications aimed at the targeted modulation of microbe–host interactions for animal production and health. In the present study, 108 content samples from six intestinal regions of dairy cows were used for shotgun metagenomic sequencing. Overall, 372 high-quality metagenome-assembled genomes (MAGs) were involved in BA deconjugation, oxidation, and dehydroxylation pathways. Furthermore, the BA-metabolizing microbiome predominately occurred in the large intestine, resulting in the accumulation of secondary unconjugated BAs. Comparative genomic analysis revealed that the bile salt hydrolase (*BSH*)-carrying microbial populations managed with the selective environment of the dairy cow intestine by adopting numerous host mucin glycan-degrading abilities. A sequence similarity network analysis classified 439 BSH homologs into 12 clusters and identified different clusters with diverse evolution, taxonomy, signal peptides, and ecological niches. Our omics data further revealed that the strains of *Firmicutes bacterium CAG-110* processed the increased abundance of BSHs from Cluster 1, coinciding with the changes in the colon cholic acid concentration after grain introduction, and were intricately related to intestinal inflammation. This study is the first to use a genome-centric approach and whole intestine-targeted metabolomics to reveal microbial BA metabolism and its diet-induced functional implications in dairy cows. These findings provide insight into the manipulation of intestinal microorganisms for improving host health.

## Introduction

Microorganisms have long been known to produce and modify biologically active metabolites that affect host health. For instance, intestinal bacteria can enzymatically modify bile acids (BAs), which act as important signaling molecules in microbe–host interactions [1]. Consequently, BAs significantly contribute to numerous physiological processes, including inflammatory responses, lipid metabolism, glucose regulation, and intestinal motility [2]. The BA transformation pathways of intestinal microorganisms vary but can be classified into four distinct categories: (1) bile salt hydrolases that mediate the deconjugation of conjugated bile salts into deconjugated BAs; (2) dehydroxylation by BA-inducible gene clusters that catalyze a multi-stage process; (3) oxidation; (4) epimerization by dehydrogenase-mediated hydroxysteroids for further modifications of various secondary BAs [3, 4]. Despite the increasing interest in identifying BA-related microbial species and enzymes in monogastric animals [5, 6], the lack of knowledge of BA metabolism in ruminants limits applications regarding the targeted modulation of microbe–host interactions for improving production and health of livestock such as dairy cows.

Dairy cows are commonly viewed as important microbe–host symbiotic paradigms that can convert low-value plant biomass into highly nutritious milk [7]. With the rapidly rising global population, the demand for dairy products is significantly increasing [8], resulting in the progressive replacement of the traditional high-forage diet with a high-grain diet [9, 10]. The economic and production benefits associated with a high-grain diet have resulted in its extensive implementation on commercial dairy farms; however, the potential disruption of the cow microbiome associated with this diet is of concern. A high-grain diet can alter physiological homeostasis, including the accumulation of fermentable carbohydrates, rumen pH, and microbial ecology [11]. These microecosystem disorders reportedly contribute to greater susceptibility to metabolic diseases in dairy cows [11]. Despite our awareness of the potential adverse effects associated with the long-term feeding of a grain-based diet on host health, previous studies have primarily focused on assessing common markers, such as volatile fatty acids (VFAs), lipopolysaccharides, lactate, and bioamines in the rumen [12, 13]. Information on BAs linking intestinal microorganisms and hosts during grain intervention in dairy cows is scarce.

In the present study, we used shotgun metagenomics to profile microbial metagenome-assembled genomes (MAGs) and targeted metabolomics to quantify BA pools from content samples of six intestinal regions in Holstein cows (universal dairy cows) fed forage-based versus grain-based diets. The specific aims of this study were to (1) decipher BA transformation pathways and their assignment to specific taxa; (2) reveal region-specific BA-metabolizing potentials from the proximal to distal intestine; (3) define BA-metabolizing gene clusters and their functional implications; (4) identify diet-induced modifications of BA-metabolizing features. Our findings lay a foundation for understanding microbial BA transformation pathways in the intestine of dairy cows, providing important clues for precise manipulation of microbial populations to improve animal health during grain feeding.

## Materials and methods

### Animals and experimental design

Animal experiments were approved by the Nanjing Agricultural University Institutional Animal Care and Use Committee (no. SYXK-2017–0027). Twelve lactating rumen-fistulated Holstein cows with a mean milk yield of 17.4 ± 4.0 kg/day and an average body weight of 651 ± 54 kg were selected and housed in individual tie stalls for the 1-month long experiment. Before animal treatment, all dairy cows were fed a forage-based diet (control, F group) with a forage:concentrate ratio of 6:4 on a dry matter (DM) basis for one week (Table S1). After the acclimation period, all dairy cows were randomly assigned to either the F (*n* = 6) or G (*n* = 6) groups. The F group continued to feed on the forage-based diet, whereas the G group was fed a grain-based diet with a forage:concentrate ratio of 4:6 on a DM basis (Table S1). This differential feeding experiment lasted 21 days, during which the animals were fed twice per day (07:00 and 19:00) ad libitum.

### Sampling scheme

Two days before the cows participating in the study were slaughtered, milk samples were collected twice daily to determine milk composition. On the last day of the experiment, all cows were slaughtered, and the internal organs were immediately dissected. The gastrointestinal tract of each cow was then separated, and the lumen contents of the rumen, duodenum, jejunum, ileum, cecum, colon, and rectum were homogenized. The pH was measured using a portable pH meter (catalog no. HI 9024C; HANNA Instruments, Woonsocket, RI, USA), and the concentrations of VFAs in the rumen and colon were determined using gas chromatography (GC-14B instrument; Shimadzu, Japan) [14]. Triplicate 5-mL samples of the homogenized contents were then collected from the six intestinal regions and stored in liquid nitrogen prior to DNA extraction. Mucosal samples from the colon were collected by scraping with a sterile slide. The mucosal tissues obtained were then stored in liquid nitrogen for subsequent RNA extraction. In addition, intact colonic tissue samples were collected and washed with ice-cold phosphate-buffered saline and cut into 0.4 × 0.4 cm sections for fixation in 4% paraformaldehyde (Sigma, St. Louis, MO, USA) for microhistomorphometry analysis.

### DNA extraction and metagenomic sequencing

Total DNA was extracted from all the intestinal content samples (~200 mg per sample) by repeated bead agitation using a microbead stirrer (Biospec Products, Bartlesville, OK, USA) [15]. The integrity of the extracted DNA was measured via electrophoresis on 0.8% agarose gels, and DNA quality and quantity were determined using a Nanodrop ND-1000 (Thermo Fisher Scientific, Waltman, MA, USA). High-quality DNA from each sample was then used to construct a metagenomic library with an insert size of 350 bp according to the manufacturer’s instructions for the TruSeq DNA PCR-Free Library Preparation Kit (Illumina, San Diego, CA, USA), and run on the NovaSeq 6000 platform (Illumina).

### Metagenome assembly

Sequence data from the intestinal microbiome were quality filtered with Trimmomatic [16] (v.0.33) to remove sequencing adapters. In addition, host, food, and human sequences were removed using BWA-MEM [17] (v.0.7.17). MEGAHIT [18] (v.1.1.1) and IDBA-UD [19] (v.1.1.3) were used to assemble high-quality reads from each sample, and the assembled contigs were combined using Minimus2 [20] (AMOS, v.3.1.0). To reduce the errors generated during assembly, BWA-ALN [21] (v.0.7.17) and SAMtools [22] (v.1.9) were applied to map all high-quality reads back to the contigs, followed by correction for single bases, insertions, and deletions. The remaining high-quality contigs were binned into MAGs using three different approaches with default parameters: MaxBin [23] (v.2.2.4), MetaBAT2 [24] (v.2.11.1), and CONCOCT [25] (v.0.4.0). The MAGs obtained were integrated using the DAS tool [26] (v.1.1.1). The completeness and contamination of prokaryotic MAGs were evaluated using CheckM [27] (v.1.0.7), and quality scores were defined as completeness −5 × contamination [28]. After filtering for completeness >80% and contamination <10%, 718 MAGs were used for predicting open reading frames in Prodigal [29] (v.2.6.3). The estimated genome size was corrected based on completeness and contamination following the algorithm from Nayfach et al. [30].

### Phylogenetic, taxonomic, and functional analyses of high-quality MAGs

All high-quality MAGs (718 MAGs identified in the present study and 260 identified in our previous study [31]) were annotated using GTDB-Tk [32] (v.0.1.6) based on the Genome Taxonomy Database. Genome properties (GPs) [33] (v.2.0) were used to characterize the functions of MAGs according to previously published studies [34]. The GPs of each MAG, classified as “Complete,” “Partial,” and “Absent” were converted into numeric values (2, 1, and 0, respectively). KofamScan (v.1.1.0) [35] was used to assign *K* numbers to the protein sequences of the 978 MAGs using HMMER/HMMSEARCH against KOfam, a customized hidden Markov model (HMM) database of Kyoto Encyclopedia of Genes and Genomes (KEGG) orthologs (KOs). The number of KO assignments with scores above the default adaptive thresholds and *E* values lower than or equal to the largest number required of KOs were considered the most reliable assignments for all search hits. The best assignments were further interpreted based on the annotation results by linking sequence data to KEGG pathways and EC numbers. Annotated results were further selected, and 15 common KOs involved in BA metabolism were retained according to previous studies [3, 36, 37]. These included choloylglycine hydrolase (or bile salt hydrolase, *BSH*), the hydroxysteroid dehydrogenases (HSDHs) *7α-HSDH*, *7β-HSDH*, *12α-HSDH*, *12β-HSDH*, *3α-HSDH*, *3β-HSDH*, 3alpha-hydroxy BA-CoA-ester 3-dehydrogenase (*baiA*), BA-coenzyme A ligase (*baiB*), 3-oxocholoyl-CoA 4-desaturase (*baiCD*), BA 7alpha-dehydratase (*baiE*), BA CoA-transferase (*baiF*), 3-dehydro-bile acid Delta4,6-reductase (*baiN*), 7beta-hydroxy-3-oxochol-24-oyl-CoA 4-desaturase (*baiH*), and BA 7beta-dehydratase (*baiI*). Based on the information available for the genes encoding these KOs, their origin in MAGs was determined and their copy numbers in the genome were calculated. The carbohydrate-active enzyme (CAZyme) profile of each MAG was predicted according to the CAZyme database [38], based on HMM using HMMER [39] (v.3.2.1). PhyloPhlAn [40] (v.1.0) was used to construct a maximum-likelihood phylogenomic tree, and iTol [41] (v.4.3.1) was used for visualization. The Enzyme Function Initiative-Enzyme Similarity Tool (EFI-EST) [42] was employed to construct a sequence similarity network (SSN) for BSH homologs, which were also predicted as signal peptides using SignalP 6.0 [43]. Finally, metaWRAP [44] (v.1.3) was used to calculate the abundance of all MAGs per sample, covering the six intestinal regions from 12 dairy cows fed forage-based and grain-based diets using the transcripts per kilobase million (TPM) calculation process.

### BA quantification

Targeted metabolomics was performed to obtain the concentrations of BA-associated metabolites in all intestinal samples. Briefly, 10 mg of each lyophilized sample was placed into an Eppendorf centrifuge tube with a safety cap. Subsequently, 25 mg of precooled grinding beads and 200 µL of mixed solvent homogenate containing 10 µL of internal standard acetonitrile/methanol (v/v = 8/2) were added to the tube. The resultant solution was then centrifuged at 4 °C and 13 500 rpm for 20 min, after which 10 µL of supernatant was collected and diluted with a 90-µL 1:1 acetonitrile/methanol (80/20) and ultra-pure water mixture. After mixing and centrifugation, 5 µL of each sample was injected for BA quantification by ultra-performance liquid chromatography–tandem mass spectrometry (UPLC-MS/MS; ACQUITY UPLC-Xevo TQ-S, Waters Corp., Milford, MA, USA) [45]. After unblinding and data release, the metabolite profiles underwent quality-control checks and data preprocessing, including batch-effect adjustment, missing value imputation, and log-transformation [46]. Data acquisition and processing were performed using MassLynx (v.4.1; Waters Corp.) [46].

### RNA extraction, library preparation, Illumina sequencing, and quantitative real-time PCR (qRT-PCR)

Total RNA was extracted from colonic mucosa samples using TRIzol reagent (Invitrogen, Carlsbad, CA, USA), following the manufacturer’s recommendations, and genomic DNA (gDNA) was eliminated using DNase I (TaKaRa Bio, Otsu, Japan). The RNA quality was measured using a 2100 Bioanalyzer (Agilent Technologies, Palo Alto, CA, USA), and the RNA concentration was quantified using the ND-2000 (Thermo Fisher Scientific). An RNA preparation kit (TruSeq RNA sample preparation Kit, Illumina Inc.) was then used to prepare 2 μg of high-quality RNA from each sample, according to the manufacturer’s instructions. Subsequently, mRNA was isolated and complementary DNA (cDNA) synthesis, end-repair, base addition, and adapter ligation were performed according to Illumina’s protocol. After PCR amplification for 200–300 bp cDNA target fragments for 15 cycles using Phusion DNA polymerase (NEB, Ipswich, MA, USA), paired-end libraries were sequenced on the NovaSeq 6000 platform (Illumina Inc.). The remaining RNA was reverse-transcribed using a PrimeScript RT Reagent Kit with gDNA Eraser (Takara Bio) for qRT-PCR.

### Transcriptome sequencing and analysis

Trimmomatic [16] (v.0.33) was used to trim raw reads and remove low-quality reads and adapters. Clean reads were then mapped onto the reference genome (no. GCA_002263795.2) in orientation mode using hisat2 [47] (v.2.2.0). The expression level of each gene transcript was estimated using the TPM method to identify differentially expressed genes (DEGs) between the F and G groups. Differential expression analysis was performed using the R package edgeR [48] (v.3.32.1). Genes with the Benjamini–Hochberg-adjusted false discovery rate (FDR) < 0.05, and log_2_ fold change (FC) > 1 were identified as DEGs. To explore the functions of the obtained DEGs, gene ontology (GO) enrichment and KEGG pathway analyses were performed using the Bioconductor GOstats package [49] and KOBAS [50] (v.3.0), respectively. In addition, genes related to the host inflammatory response (*ITIH4*, *LBP*, *HP*, *SAA4*, *SAA2*, *F2*, *AHSG*, and *SERPINF2*) were further validated by quantitative PCR, and a *t*-test was used to statistically compare the qRT-PCR results. The primer sets used are listed in Table S2.

### Statistical analyses

The abundances of MAGs and BSH proteins were compared between the F and G groups using the Wilcoxon rank-sum test. For BA composition, each metabolite was compared between the two diet groups using a *t*-test when the metabolites of each group were normally distributed (Sharipo test, *p* > 0.05) and showed homogeneity of variance (Levene test, *p* > 0.05). Otherwise, metabolites were compared using the Wilcoxon rank-sum test. The obtained *p* values of MAGs and BSH proteins between the two diet groups were then used to correct the FDR based on the Benjamini–Hochberg procedure using R with the parameter “p.adjust”.

## Results

### MAGs are involved in BA transformation pathways in the intestine of dairy cows

**Fig. 1:**
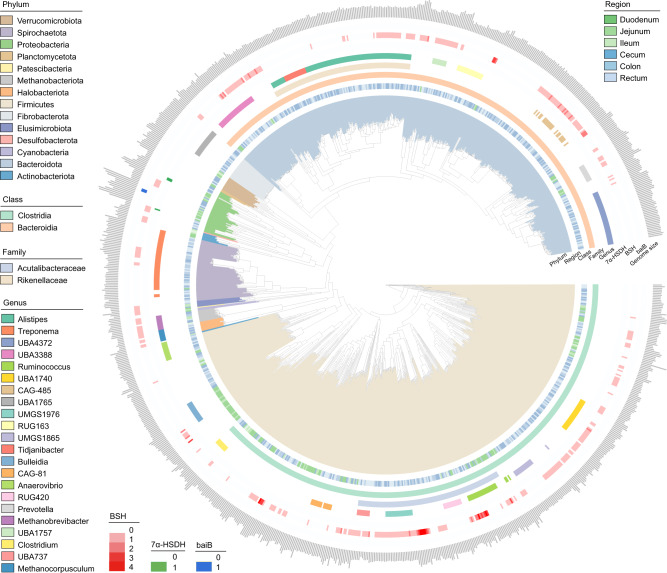
Phylogenetic tree of 978 metagenome-assembled genomes (MAGs) sampled from the intestine of dairy cows. The clades are colored according to phylum. The heat map represents the MAGs from different intestinal regions. The three strip charts indicate the class, family, and genus-level affiliations of MAGs. The next three strip charts indicate the number of *7α-HSDH* (7-alpha-hydroxysteroid dehydrogenase [EC:1.1.1.159]), *BSH* (bile salt hydrolase [EC:3.5.1.24]), and *baiB* (bile acid-coenzyme A ligase [EC:6.2.1.7]) in each MAG. The outer layer of the bar graph represents genome size.

Shotgun metagenomic sequencing was performed for 72 content samples covering six intestinal regions from 12 dairy cows, including the duodenum, jejunum, ileum, cecum, colon, and rectum. Over 1.6 Tb of data enabled the recovery of 718 high-quality prokaryotic MAGs, all of which were >80% complete with <10% contamination evaluated by CheckM. By recruiting an additional 260 high-quality MAGs (completeness >80% and contamination <10%) obtained from 36 intestinal samples from six cows in our previous study [31], a total of 978 high-quality MAGs from 18 dairy cows were examined (Table S3). All 978 MAGs were primarily represented by the families *Acutalibacteraceae* (106) and *Rikenellaceae* (102; primarily *Alistipes* spp.; Fig. 1; Table S4).

For annotation analysis, 372 MAGs (over 38%) involved in BA transformation pathways were identified, including deconjugation, oxidation, and dehydroxylation (Fig. 1; Table S5). Of these, 368 MAGs encoded *BSH*, which hydrolyzes conjugated bile salts into deconjugated BAs (Fig. 1). These MAGs were assigned to nine phyla, including *Firmicutes* (185) and *Bacteroidetes* (135; Fig. 1), indicating that multiple phylotypes are capable of bile salt hydrolysis in the intestine of dairy cows. These *BSH*-carrying MAGs primarily belonged to families *Acutalibacteraceae* (18.8%), *CAG-272* (12.8%), *Muribaculaceae* (10.3%), *Rikenellaceae* (7.3%), and *Lachnospiraceae* (5.7%), and predominately assigned to genus *Alistipes* (26) and *CAG-485* (20; Fig. 1, Table S5). In addition, we determined that only three MAGs encoded *7α-HSDH* for oxidizing the hydroxyl group of deconjugated BAs in an NAD(P)^+^-dependent manner; these MAGs were *Escherichia flexneri* (MAG172 and MAG373) and *Yoonia* sp. (MAG697; Fig. S1A, B; Table S5). Only two MAGs belonging to *BOG-935* (*Alphaproteobacteria*) were *baiB*-containing strains, which contributes to CoA ligation in an ATP-dependent manner (Fig. S1C, D; Table S5). Therefore, only a few bacteria inhabiting the intestine of dairy cows are capable of secondary BA modification.

### BA metabolism of intestinal microorganisms specific to dairy cows

**Fig. 2:**
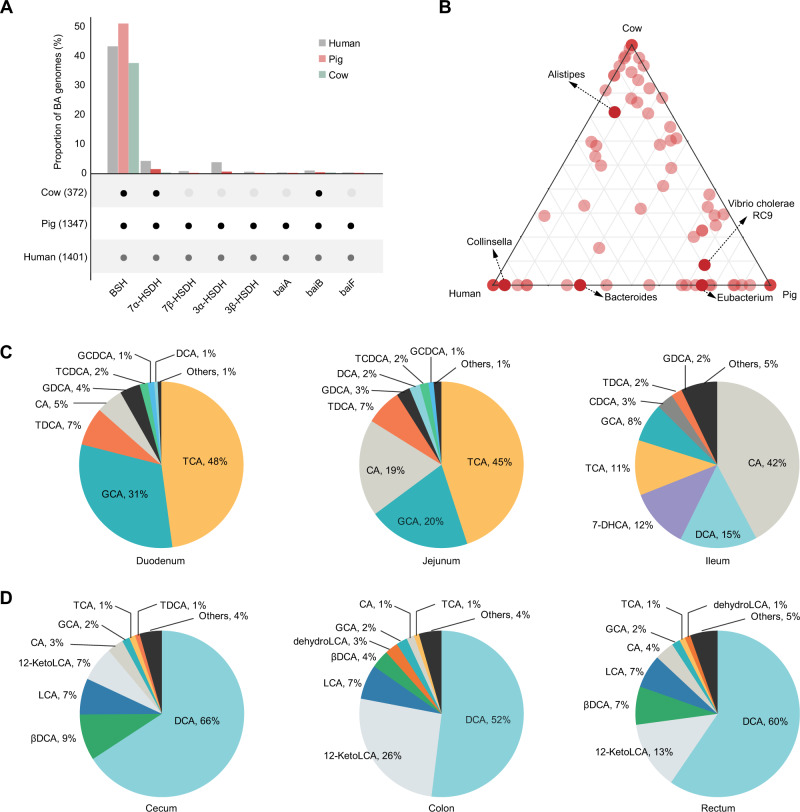
Bile acid metabolism of intestinal microbiota specific to dairy cows. **A** Comparison of the genes involved in bile acid metabolism in the intestine of dairy cows, humans, and pigs. **B** Classification of *BSH*-carrying MAGs at the genus level in the intestine of dairy cows, humans, and pigs. BSH, bile salt hydrolase; HSDH, hydroxysteroid dehydrogenase; *bai*, bile acid-inducible. **C** Proportions of bile acids in the small intestine of dairy cows. **D** Proportions of bile acids in the large intestine of dairy cows.

The BA-metabolizing specificity of microorganisms in the intestine of dairy cows was captured by comparing the 978 high-quality intestinal MAGs obtained here with those of humans (2 935 MAGs) [51] and pigs (2 564 MAGs) [52] reported in previous studies (Table S6). Our taxonomic and functional annotations revealed that the intestinal MAGs of humans and pigs harbored *BSH*, *7α-HSDH*, *7β-HSDH*, *3α-HSDH*, *3β-HSDH*, *baiA*, *baiB*, and *baiF*, which are involved in BA transformation pathways, whereas the intestinal MAGs of dairy cows lacked *7β-HSDH*, *3α-HSDH*, *3β-HSDH*, *baiA*, and *baiF* (Fig. 2A).

Regarding BA deconjugation, *BSH* was widespread in a host-independent manner in 43.1% of the human intestinal MAGs, 51.0% of the pig intestinal MAGs, and 37.6% of the dairy cow intestinal MAGs (Fig. 2A). At the family level, *BSH*-carrying MAGs were primarily assigned to *Acutalibacteraceae* in dairy cows (18.8%) and pigs (14.3%), whereas *Lachnospiraceae* (16.7%) was the main *BSH*-carrying family in the human intestine (Tables S5 and S6). *Alistipes* (7.1%) was the top *BSH*-carrying genus in the intestine of dairy cows but it accounted for a lower proportion of *BSH*-carrying MAGs in humans and pigs (Fig. 2B). Overall, BA transformation pathways of intestinal microorganisms were driven by heterologous populations specific to distinct host species. The higher proportions of family *Acutalibacteraceae* and genus *Alistipes* with bile salt hydrolysis ability may be adaptive characteristics of the dairy cow intestine in response to associated environmental conditions.

Among all the encoded HSDHs, *7α-HSDH* was the most widespread in the intestine of monogastric animals, accounting for 4.02% of MAGs in humans and 1.48% in pigs (Fig. 2A). The *bai*-carrying MAGs involved in the multistep 7α-dehydroxylation pathway of primary BA accounted for <1% in both human and pig (Fig. 2A). Notwithstanding, the intestinal microorganisms of dairy cows had a lower participation in the modification process of secondary BAs compared to monogastric animals.

### Region-specific BA-metabolizing potential along the intestine of dairy cows

The taxonomic populations and BA profiles were compared to systemically characterize the BA-metabolizing potentials in the six intestinal regions of dairy cows. Over 88.4% of the BA-metabolizing MAGs were obtained from the large intestine (cecum, 104; colon, 103; rectum, 122), followed by 21, 18, and 4 MAGs from the duodenum, jejunum, and ileum, respectively (Fig. 1; Table S5). Most of the BA-metabolizing MAGs from the duodenum and jejunum were classified into classes *Clostridia* (57.1% and 38.9%, respectively) and *Bacilli* (19.1% and 16.7%, respectively). The BA-metabolizing MAGs from the ileum belonged to classes *Actinomycetia* (*Bifidobacterium globosum*, 50%), *Methanobacteria* (25%), and *Clostridia* (25%) (Table S5). In the large intestine, the classes *Bacteroidia* and *Clostridia* accounted for over 85% of the BA-metabolizing MAGs (Table S5). Overall, the different taxonomic groups associated with BA metabolism showed a region-specific distribution from the proximal to distal intestine, and microorganisms in the large intestine contribute significantly to BA metabolism in dairy cows.

Targeted metabolomics was further conducted using UPLC-MS/MS to quantify the BA profiles in the six intestinal regions. From this analysis, 27 BA species were obtained, and variations were observed in the concentrations and relative compositions of these species among the six intestinal regions (Fig. 2C, D; Table S7). The concentrations of conjugated BAs were highest in the duodenum and jejunum, followed by the ileum, and finally the large intestine. These BAs included taurocholic acid (TCA), glycocholic acid (GCA), taurodeoxycholic acid, glycodeoxycholic acid, taurochenodeoxycholate, glycochenodeoxycholic acid, taurolithocholic acid, and glycolithocholic acid (Table S7). The primary unconjugated BAs in the duodenum and jejunum were TCA and GCA. TCA accounted for 47.9% and 44.9% of all BAs detected in the duodenum and jejunum, respectively. However, TCA was far less prevalent in the large intestine (1.1% in each region; Fig. 2C, D). Approximately 98.8% of BAs were efficiently reabsorbed into the bloodstream in the ileum (Table S7). In contrast, secondary unconjugated BAs had higher concentrations in the large intestine via microbial transformation pathways, such as βDCA, LCA, 12-KetoLCA, isoLCA, and isoalloLCA (Table S7). Among them, DCA accounted for the largest portion of all measured BAs in the large intestine, whereas it only accounted for 0.67% and 2.1% in the duodenum and jejunum, respectively (Fig. 2C, D). Therefore, conjugated BAs constituted the largest portion in the duodenum and jejunum and were efficiently absorbed in the ileum, followed by the enrichment of microbe-derived secondary unconjugated BAs in the large intestine.

### Functional traits of *BSH*-carrying MAGs along the intestine of dairy cows

**Fig. 3:**
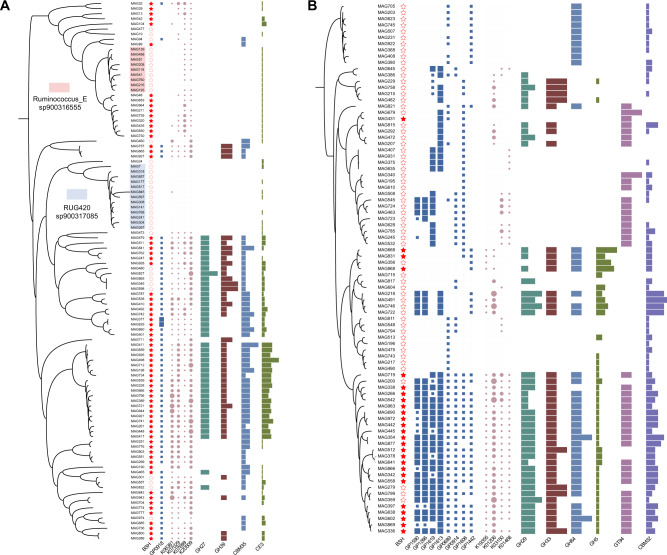
Functional advantages of bile salt hydrolase (*BSH*)-carrying metagenome-assembled genomes (MAGs) in the intestine of dairy cows. Comparison of the functional differences in genome properties, Kyoto Encyclopedia of Genes and Genomes (KEGG) orthologous groups, and carbohydrate-active enzymes between *BSH*-carrying and non-*BSH*-carrying MAGs belonging to the family *Acutalibacteraceae* (**A**) and genus *Alistipes* (**B**). GenProp0918 (GP0918), Anaerobic sulfatase/maturase system; GP1590, 4-Amino-2-methyl-5-diphosphomethylpyrimidine biosynthesis; GP1266, Superpathway of thiamine diphosphate biosynthesis II; GP1619, L-histidine degradation I; GP1613, Formate assimilation into 5,10-methylenetetrahydrofolate; GP0689, Glyoxalate conversion to phosphoglycerate; GP0914, [FeFe]-dependent hydrogenase; GP1606, Octopamine biosynthesis;GP1442, Protein O-[N-acetyl]-glucosylation; K06381, spoIID, stage II sporulation protein D; K07029, dagK, diacylglycerol kinase (ATP); K07699, spo0A, stage 0 sporulation protein A; K20509, madB, carboxybiotin decarboxylase; K19355, MAN, mannan endo-1,4-beta-mannosidase; K01206, FUCA, alpha-L-fucosidase; K03150, thiH, 2-iminoacetate synthase; K01468, hutI, imidazolonepropionase.

The initial “gateway reaction” is the deconjugation of primary BAs employed by *BSH*, which mediates bile tolerance to adapt to intestinal selective stress [3, 53]. Therefore, the functional potential of the *BSH*-carrying MAGs in the intestine of dairy cows was explored by comparing representative consortia of the family *Acutalibacteraceae* and genus *Alistipes* that encode *BSH* with those that do not. Comparing the functional profiles obtained from GPs, KOs, and CAZymes revealed that the *BSH*-carrying MAGs (BCGs) assigned to *Acutalibacteraceae* had a prevalence of GH27 and GH39 involved in alpha-galactosidase, alpha-*N*-acetylgalactosaminidase, and alpha-L-iduronidase (Fig. 3A), thereby contributing to the degradation of host-derived mucin glycans. Anaerobic sulfatase maturases were encoded in the *Acutalibacteraceae* BCGs to degrade sulfated O-glycans from intestinal mucin but were absent from the non-*BSH*-carrying MAGs (NCGs; species *Ruminococcus_E sp900317315* and *RUG420 sp900317085*; Fig. 3A). Another unique protein in the BCGs assigned to *Acutalibacteraceae* but absent from the NCGs was the Stage II sporulation protein D (*SpoIID*; Fig. 3A), a lytic transglycosylase essential for sporulation. All *Alistipes* BCGs contained numerous alpha-L-fucosidase (GH29) and sialidase (GH33), whereas most *Alistipes* NCGs lacked the CAZymes required to degrade host mucin glycans (Fig. 3B). In addition, a general enrichment of GPs associated with 4-amino-2-methyl-5-diphosphomethylpyrimidine biosynthesis (GenProp1590) and superpathways of thiamine diphosphate biosynthesis II (GenProp1266) in *Alistipes* BCGs was observed. Conversely, *Alistipes* NCGs lacked these thiamine biosynthesis pathways (Fig. 3B). These findings suggest that *BSH*-carrying microorganisms have strong functional advantages in the intestine of dairy cows, including nutrient substrate competitiveness, resistance to external environmental conditions, and metabolic ability.

To further explore the adaptive traits of *BSH*-carrying microorganisms to distinct niches from the proximal to distal intestine, their taxonomic and functional properties in the six intestinal regions were compared. Among the taxonomic populations, *Erysipelotrichaceae*-affiliated MAGs were distributed in the small intestine (primarily in the duodenum, followed by the jejunum), whereas the large intestine was populated by a large number of *Bacteroidales*- and *Oscillospirales*-affiliated MAGs. Profiling the carbohydrate-related gene properties revealed that *BSH*-carrying MAGs from the small intestine selectively processed lysozyme (GH25) to utilize microbe-derived peptidoglycan, whereas those (particularly *Bacteroidales*) from the large intestine processed GH23, GH73, and CBM50 for peptidoglycan degradation (Table S8). In addition, the hindgut microbiome preferentially utilized host-derived mucin glycans and xylans, including the enrichment of alpha-*N*-acetylgalactosaminidase GH109 in *Clostridiales bacterium UBA1740*-affiliated MAGs and the representation of acetyl xylan esterase CE3 in *UMGS1976* (Table S8). In addition, the small intestine-enriched MAGs, particularly *Erysipelotrichaceae*, had a higher representation of membrane transport systems, including oligopeptide transporters (*OppABC*), oligosaccharide transporters (*msmX*), amino acid transporters (*metNIQ*), nucleoside transporters (*nupABC*), and phosphotransferase transport systems (Tables S9 and S10). In contrast, the pyruvate to acetyl-CoA pathway for further fermentation was enriched in the large intestine via *korAB* (Table S9). Therefore, regional nutrient availability is an important filter for selecting microbial populations with specific functions.

### Classification and variation patterns of BSHs in the intestine of dairy cows

**Fig. 4:**
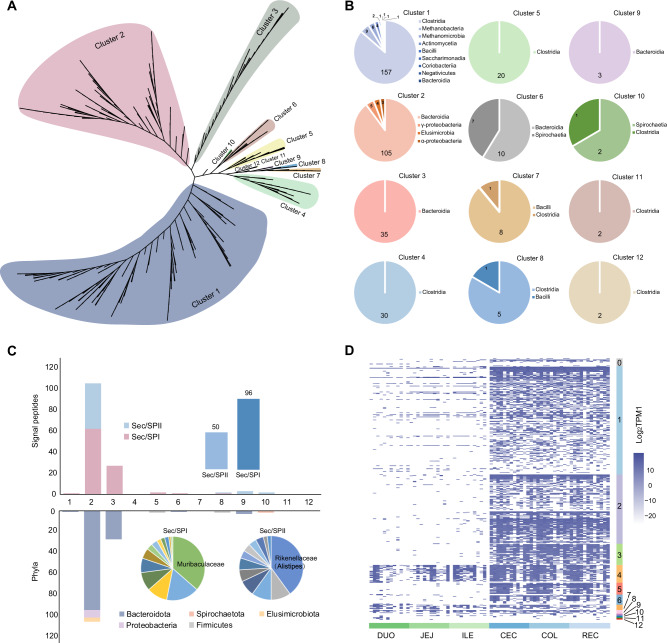
Classification and variation of 439 bile salt hydrolase (BSH) homologs in the intestine of dairy cows. **A** All 12 clusters of BSHs based on a sequence similarity network (>40% sequence identity). **B** Taxonomic characterization of each cluster at the class level. **C** Prediction of signal peptides for the 439 BSH proteins among different clusters. Sec/SPI, Sec signal peptides; Sec/SPII, lipoprotein signal peptides. **D** Heatmap of the abundance and distribution of 12 BSH clusters from six intestinal regions in 12 dairy cows, including duodenum, jejunum, ileum, cecum, colon, and rectum. TPM1, TPM + 0.0000001; DUO duodenum; JEJ jejunum; ILE ileum; CEC cecum; COL colon; REC rectum.

The broad range of sequence dissimilarity in BSHs among multiple phylogenetically diverse strains [54, 55] prompted us to use EFI-EST [42] to construct an SSN with 439 BSH homologs from 368 *BSH*-carrying MAGs in the intestinal microbiome of dairy cows. Our alignment score threshold setting allowed classification of all BSH homologs into 12 clusters (>40% sequence identity; Fig. 4A). Cluster 1 contained 182 BSH homologs from 172 MAGs, which were assigned to *Clostridia* (86.3%), *Methanobacteria* (4.9%, species *Methanobrevibacter sp900314635*), *Methanomicrobia* (3.3%, *Methanocorpusculum* spp.), *Actinomycetia* (2.2%, *Bifidobacterium globosum*), and *Bacilli* (1.1%; Fig. 4B). Cluster 2 contained 117 BSH homologs from 112 MAGs, including *Bacteroidia* (89.7%, primarily *Alistipes* spp.), *Gammaproteobacteria* (4.3%), *Elusimicrobia* (3.4%), and *Alphaproteobacteria* (2.6%; Fig. 4B; Table S11). The sequences of Cluster 3 (35 BSH homologs) and Cluster 9 (three BSH homologs) were all encoded by *Bacteroidia* (Fig. 4B). *Clostridia* were assigned to Cluster 4 (30 BSH homologs, primarily *Ruminococcus_E sp900316555*), Cluster 5 (20 BSH homologs), Cluster 8 (five BSH homologs), Cluster 11 (two BSH homologs), and Cluster 12 (two BSH homologs; Fig. 4B). Seventeen BSH homologs in Cluster 6 were affiliated with *Bacteroidia* (58.8%) and *Spirochaetia* (41.2%; Fig. 4B). For Cluster 7, *Bacilli* (family *Erysipelotrichaceae*) contributed to eight BSH homologs and *Clostridia* to one BSH homolog (Fig. 4B). The three BSH homologs in Cluster 10 belonged to *Spirochaetia* and *Clostridia* (Fig. 4B).

The signal peptides of the 439 BSH proteins among the different clusters, which are short *N*-terminal amino acid sequences that control protein secretion and translocation [43], were also predicted. Approximately 33.3% (146) of the BSH homologs contained signal peptides, including 96 Sec signal peptides (Sec/SPI) and 50 lipoprotein signal peptides (Sec/SPII; Fig. 4C; Table S12). Notably, signal peptides were primarily predicted from BSH sequences in Clusters 2 and 3, almost all from class *Bacteroidota* (Fig. 4C). Of all the signal peptides, Sec/SPI was most frequently present in *UBA3388 sp002358835* from Cluster 2 and family *Muribaculaceae* from Cluster 3, while 40.0% of the BSH sequences from *Alistipes* spp. in Cluster 2 contained Sec/SPII (Fig. 4C; Table S12). It was also observed that only BSH homologs from Clusters 4, 7, and 8 were prevalent in the small intestine, whereas BSH homologs from Clusters 1 to 8 were enriched in the large intestine (Fig. 4D). These results indicate that different BSH homologs exhibit sequence dissimilarity, which may lead to variable functional implications in the deconjugation of BAs in the intestine of dairy cows.

### Modification of the microbiome signature from grain-based diet introduction

The functional implications of microbial BA metabolism in forage-based (control) and grain-based diets were further explored, as these are two common dietary regimens used in dairy cow production. The reduced percentage of milk fat was the main production performance feature in the grain-based diet intervention (*t*-test, *p* = 0.019); however, no significant changes were observed in the percentage of milk protein and lactose between the two groups (Fig. S2). Significant changes caused by diet intervention were also reflected in the fermentation parameters, such as the reduction in pH and increased concentrations of propionate, butyrate, and valerate in the rumen, followed by increased concentrations of propionate and valerate in the colon (*t*-test, *p* < 0.05; Fig. S3).

**Fig. 5:**
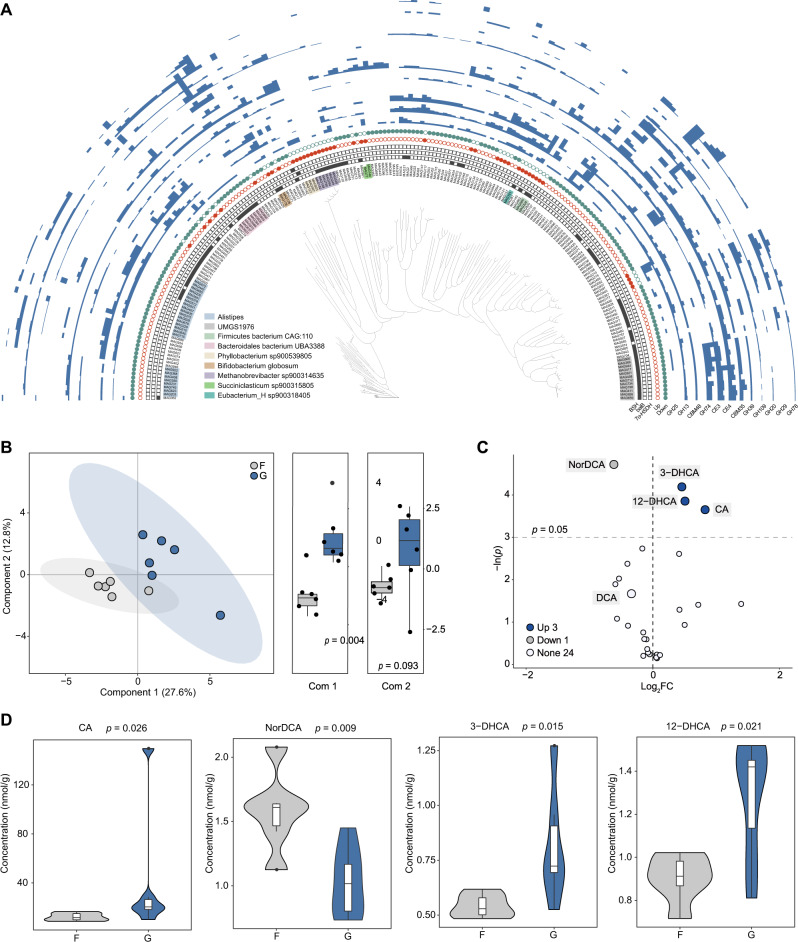
Changes in the microbial composition and bile acid (BA) profiles in the colon of dairy cows after grain intervention. **A** Altered abundance of metagenome-assembled genomes (MAGs), including BA-metabolizing MAGs and their associated CAZyme profiles. **B** PLS-DA 2D score plot with a principal component boxplot between the forage-based (F) and grain-based (G) diets. **C** Volcano plot of 28 BA-associated metabolites in the colon of dairy cows in F and G diet groups. The blue dots represent increased BAs (FDR < 0.05), and the gray dots represent decreased BAs (FDR < 0.05). CA, cholic acid; NorDCA, 23-nordeoxycholic acid; 3-DHCA, 3-dehydrocholic acid; 12-DHCA, 12-dehydrocholic acid; DCA, deoxycholic acid. **D** Violin plot of all significantly differential BAs between the F and G diets.

Recognizing that colon microbiota is major agent of BA transformations important for host health or disease [56], the colonic microbiome signature in the forage-fed and grain-fed cows was also analyzed, and significant differences in the microbial composition were found between these two groups (ANOSIM, *p* = 0.002; Fig. S4A). Compared with the forage-fed cows, the grain-based diet caused significant changes in 206 MAGs, including the increased abundance of 43 MAGs and decreased abundance of 163 MAGs (Wilcoxon rank-sum test, FDR < 0.05; Fig. 5A). The increased MAGs primarily belonged to *Methanobrevibacter sp900314635*, *Phyllobacterium sp900539805*, *Bifidobacterium globosum*, and propionate-producing bacteria *Succiniclasticum sp900315805*. The abundance of MAGs belonging to *Methanobrevibacter sp900314635* and propionate-producing bacteria *Succiniclasticum sp900315805* had over 1.7-fold increase. More than ten-fold increased abundance was observed in the MAGs belonging to *Phyllobacterium sp900539805* and *Bifidobacterium globosum*. In contrast, the reduced-abundance MAGs were predominately assigned to *Alistipes* spp. and *UMGS1976* spp. (*Acutalibacteraceae*; over 1.5-fold decrease). Further comparison of the CAZyme profiles showed that the increased MAGs were predicted to process lysozyme (GH25) to break down the bacterial cell walls.

The BA-metabolizing microbiome was also significantly affected by the grain-based diet, compared with the forage-based diet (ANOSIM, *p* = 0.002; Fig. S4B). Differential abundances in the colon of the grain-fed versus the forage-fed cows were also observed (Wilcoxon rank-sum test, FDR < 0.05, FC > 1.5; Fig. 5A). The increased BA-metabolizing MAGs after grain-based diet introduction were primarily affiliated with *Methanobrevibacter sp900314635*, *Bifidobacterium globosum*, *Firmicutes bacterium CAG-110*, and *Eubacterium_H sp900318405*. Among them, *Bifidobacterium globosum* (MAG178 and MAG176) and *Firmicutes bacterium CAG-110* (MAG324) showed a prevalence of alpha-amylase (GH13 and CBM48; Fig. 5A). In addition, the grain-based diet reduced MAGs abundance, primarily for *UMGS1976* (family *Acutalibacteraceae*), *Bacteroidales bacterium UBA3388*, and *Alistipes*. These decreased MAGs mostly encoded enzymes associated with cellulose and hemicellulose degradation (GH74, CE3, CE4, CBM35, and GH39), specifically the *UMGS1976*-affiliated MAGs (Fig. 5A). In addition, the enrichment of host mucin glycan-degrading enzymes was another feature of reduced MAGs, including GH109, GH20, GH29, and GH78 (Fig. 5A). Therefore, these results highlight that changes in BA-metabolizing MAGs are accompanied by the altered microbial substrate in dairy cows fed the forage-based and grain-based diet.

### Reconstruction of microbial BA profiles and host inflammatory response in the colon by grain intervention

The BA profiles of forage-fed and grain-fed cows were compared using partial least squares discriminant analysis, and the first PLS component was found to be significantly different between groups (*p* = 0.004; Fig. 5B). The concentrations of four BAs varied notably between the forage and grain-fed groups (Fig. 5C). The grain-based diet caused a significant increase in cholic acid CA (Fig. 5D; Wilcoxon rank-sum test, *p* = 0.026), suggesting the accumulation of primary unconjugated BAs. The concentrations of 3-dehydrocholic acid (3-DHCA; Wilcoxon rank-sum test, *p* = 0.015) and 12-DHCA (*t*-test, *p* = 0.021) were also elevated by the grain-based diet, whereas the concentration of 23-nordeoxycholic acid (NorDCA; *t*-test, *p* = 0.009) was reduced (Fig. 5D). Given the accumulation of primary unconjugated BAs, the changes in the abundance of BSH homologs in the colon after grain introduction were also determined, and notable differences were observed in the abundance of 46 BSHs (increases in five BSHs and decreases in 41 BSHs; Wilcoxon rank-sum test, FDR < 0.05; Fig. S5). All BSHs with increased abundance were from Cluster 1, lacked signal peptides, and were found in *Oscillospirales* (Table S13), including MAG560, MAG324, MAG281, MAG847, and MAG538. Of these, MAG560 and MAG324 were assigned to *Firmicutes bacterium CAG-110*, whose abundance was significantly increased after grain introduction (Fig. 5A). In contrast, BSHs with decreased abundance were affiliated with Clusters 2 (17 BSH homologs), 4 (8 BSH homologs), 5 (17 BSH homologs), and 7 (3 BSH homologs), and ~40% of them contained signal peptides (Table S13).

**Fig. 6:**
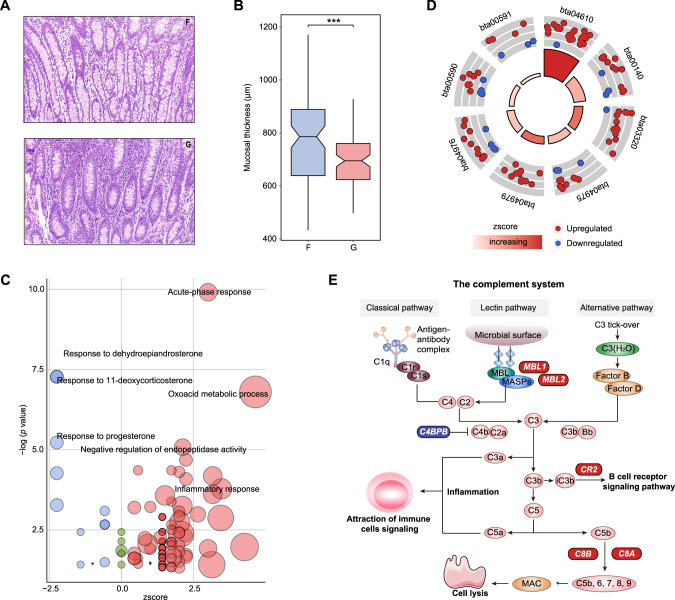
Reconstruction of the host inflammatory response in the colonic mucosa after grain introduction. Histology (**A**) and mucosal thickness (**B**) of the colon. **C** Gene ontology (GO) analysis based on differentially expressed genes (DEGs). Upregulated GO terms are in red; unchanged GO terms are in green; downregulated GO terms are in blue. **D** Kyoto Encyclopedia of Genes and Genomes (KEGG) pathway enrichment analysis of DEGs. bta04610, Complement and coagulation cascades; bta00140, Steroid hormone biosynthesis; bta03320, PPAR signaling pathway; bta04975, Fat digestion and absorption; bta04979, Cholesterol metabolism; bta04976, Bile secretion; bta00590, Arachidonic acid metabolism; bta00591, Linoleic acid metabolism. **E** DEGs related to complement system. MAC, membrane attack complex. Significantly different genes encoding enzymes involved in the related pathways are shown in red (upregulation in the grain-fed animals) and blue (downregulation in the grain-fed animals). z score = (up-down)/count^(1/2), wherein, up, down, and count respectively represent the number of upregulated, downregulated, total DEGs in related GO or KEGG terms.

Morphological analysis and transcriptome sequencing of colonic mucosa samples from dairy cows in both diet groups were further performed. The histological analysis of the colon indicated that the grain-based diet increased mucosal lymphocytes and led to an irregular arrangement of the intraepithelial mucous gland (Fig. 6A), followed by a decrease in mucosal layer thickness (*t*-test, *p* < 0.001; Fig. 6B). A total of 18,579 genes were obtained with an average expression >0.5 TPM in at least one group. Among the available encoded genes, 723 DEGs were observed, including 488 upregulated and 235 downregulated genes, for the grain-based diet group (edgeR, Benjamini–Hochberg adjusted FDR < 0.05 and log_2_FC > 1). GO enrichment analysis was further conducted based on the biological processes and a significant increase in acute-phase proteins associated with the inflammatory response of colonic mucosa was observed for the grain-based diet group (Fig. 6C). These proteins included haptoglobin precursor (HP), serum amyloid A (SAA2 and SAA4), lipopolysaccharide-binding protein precursor (LBP), prothrombin isoform X1 (F2), alpha-2-antiplasmin precursor (SerpinF2), alpha-2-HS-glycoprotein precursor (AHSG), and inter-alpha-trypsin inhibitor heavy chain H4 isoform X1 (ITIH4). KEGG pathway analysis revealed that most DEGs were enriched in complement and coagulation cascades (Fig. 6D, E), resulting in a host inflammatory response. Of them, mannose-binding lectin (MBL) precursors (MBL1 and MBL2) were upregulated in the grain-fed dairy cows to activate the lectin pathway (Fig. 6E). The downregulated expression of C4b-binding protein beta chain isoform X1 (C4BPB) promoted the formation of C3 convertase by attenuating inhibition (Fig. 6E). The upregulated expression of complement receptor type 2 precursor (CR2) activated the B cell receptor signaling pathway (Fig. 6E). In addition, the grain-based diet elevated the expression of complement component C8 chain precursors (C8A and C8B) to promote the formation of membrane attack complex (MAC), ultimately resulting into cell lysis (Fig. 6E). The mRNA expression of the targeted genes was further validated by qRT-PCR, and the expression trends remained consistent (Fig. S6). Overall, the grain-based diet led to an inflammatory response in colonic mucosa.

## Discussion

The BA transformation pathways of microorganisms within six intestinal regions were determined from 108 content samples obtained from 18 dairy cows. Our genome-centric approach revealed that over 38% of the microbial population participated in BA deconjugation, oxidation, and dehydroxylation pathways within the intestine of dairy cows. Further integration with targeted BA pools revealed that the BA-metabolizing microbiome primarily originated from the large intestine, resulting in the accumulation of secondary unconjugated BAs in dairy cows. These microbiota-derived unconjugated BAs play an indispensable role in intestinal immunity and inflammation [37, 57]. In contrast, liver-derived primary conjugated BAs were enriched in the duodenum and jejunum of dairy cows. Among them, primary conjugated BAs were primarily conjugated with taurine in dairy cows, which is more similar to mice than humans [56]. The representation of these host-derived conjugated BAs in the duodenum and jejunum facilitates dietary lipid and vitamin absorption [37]. To the best of our knowledge, the present study is the first to use a genome-centric approach and whole-intestine targeted metabolomics to examine microbial BA metabolism in ruminants. Our findings will contribute to future studies on manipulating BA pools to improve ruminant production and health.

Bile tolerance is a crucial property of microbial consortia as it determines the ability of a strain to survive in the intestine. From a host-specific perspective, our study showed a higher prevalence of bile salt hydrolysis ability in microbial populations assigned to the family *Acutalibacteraceae* and genus *Alistipes* in dairy cows than in human or pigs. Notably, these microbial populations cope with stressful intestinal conditions through bile tolerance and by adopting numerous capabilities for host mucin glycan degradation, sporulation, and thiamine biosynthesis. For example, the *BSH*-carrying *Alistipes* spp. participated in thiamine biosynthesis. Thiamine is an essential cofactor for energy, amino acid, and nucleotide metabolism that is primarily biosynthesized by hindgut microorganisms in ruminants [58, 59]. A previous study reported that *Alistipes* spp. have relatively low abundance and are new to the human intestine, with emerging implications for inflammation, cancer, and mental health [60]. However, these bacteria accounted for 8.7% of the MAGs in the large intestine of dairy cows (Table S1). Therefore, *Alistipes* spp. with bile tolerance ability seems to play an important role in the hindgut niche.

In addition, it was determined that *BSH*-carrying populations possessed innovative nutritional strategies for mucosal health. The degradation process used by intestinal mucin-degrading microbes is complex and includes the terminal residues on the O-glycans (sialic acid, fucose, and glycosulfate) as the first targets [61, 62], followed by sequential removal of the released terminal sugars [63] and further hydrolysis of the core structures of O-glycan chains when the peripheral residues have been removed [64]. The *BSH*-carrying populations found in the dairy cow intestine were enriched in fucosidases, sialidases, and sulfatases that would allow for the removal of these modifications, thereby releasing the terminal sugars and exposing O-glycan chains for use by cross-feeding microorganisms. This nutrient-based cross-feeding or cooperative resource-sharing highlights the complex food chains that specific microbial consortia with bile tolerance have formed for harvesting ubiquitously abundant host-derived mucin glycans and for survival in the intestinal environment as a stable microbial network in the intestine of dairy cows.

Regional differences in the functional properties of *BSH*-carrying populations were observed from the proximal to distal intestine of dairy cows. In the large intestine, the enrichment of anaerobic *Bacteroidales* and *Oscillospirales* contributes significantly to the fermentation process [65]. In contrast, most members of *Erysipelotrichaceae* exhibit aerobic respiration and were distributed in the small intestine. Additionally, the *BSH*-carrying MAGs from the small intestine were responsible for the uptake of simple nutrients directly, whereas the hindgut-enriched microorganisms tended to utilize host mucin glycans. Host-secreted mucin glycans are important substrate sources for intestinal microorganisms, particularly in the distal intestine [62]. Therefore, regional microbial consortia with bile tolerance have great diversity to adapt to different niches, such as different oxygen levels, nutrient bioavailability, and host mucus composition.

Our study further highlights the diversity and divergence of the bile salt hydrolysis mechanisms. Our SSN classified 439 BSH homologs from the intestinal microbiome of dairy cows into 12 clusters. In contrast to sequence alignment in phylogenetic analysis, SSN can reveal sequence-structure-function associations and evolutionary relationships among different protein families [42, 55, 66]. This taxonomic lens revealed that the different clusters were mostly driven by distinct genera, such as *Alistipes* in Cluster 2, *Ruminococcus* in Cluster 4, and *Treponema* in Cluster 6 (Table S11), indicating that the 12 clusters may have evolved from different hydrolase precursors [55]. In addition, the classification system separated BSH homologs with and without signal peptides. Most enzymes in Clusters 2 and 3 contained N-terminal signal peptides, whereas those in the other clusters lacked these peptides. Although the relationship between the presence or absence of signal peptides and the hydrolysis activity of bile salts is unclear, signal peptides may drive hydrolysis in different ways [43, 67]. Based on the presence of signal peptides, it is speculated that most BSH enzymes from Clusters 2 and 3, which were affiliated with the class *Bacteroidia*, were secretory proteins, while those from the class *Clostridia* were not. Furthermore, the diverse BSH clusters reflected different ecological niches in the intestine of dairy cows. For instance, the representation of Clusters 4, 7, and 8 in the small intestine emphasized the close relationship between these BSH homologs and small intestine health. In contrast, the BSH homologs from Clusters 1 to 8 contributed to hindgut health. Overall, our findings regarding the classification of BSHs based on large-scale SSN provide new insights into the evolution, function, and ecology of BSH homologs in the intestine of dairy cows.

The complex association between BA metabolism and intestinal health in dairy cows was further assessed. Evidence from monogastric animals has shown that microbe-derived BAs act as important signaling molecules to modulate interactions between intestinal microorganisms and their host, including host metabolic and inflammatory responses [1, 3, 54]. However, knowledge on BAs acting as links between microorganisms and host health during grain intervention in ruminants is scarce. Our targeted metabolomic analysis established several differences in the BA profiles of dairy cows fed two common dietary regimes, with the most striking differences observed for CA, a primary unconjugated BA, which was significantly elevated in grain-fed cows. Moreover, a grain-based diet significantly reshaped the composition of BA-metabolizing MAGs, which also favored starch degradation by reducing plant biomass hydrolysis and host mucin glycan degradation. The reduced host glycan-degrading microbiota suggests the depletion of microbiota-accessible glycans in the colonic mucus layer, which coincided with the decreased mucosal thickness after grain intervention. Among the increased abundance of BA-metabolizing MAGs, we noted that the strains of *Firmicutes bacterium CAG-110* processed the elevated abundance of BSHs from Cluster 1, consistent with the alteration of CA concentration. Our previous study reported the hub consortia of *Firmicutes bacterium CAG-110* in the large intestine of ruminants [31], highlighting its indispensable role in the accumulation of primary unconjugated BAs in the colon after grain intervention. Previous studies have shown that the accumulation of primary unconjugated BAs and BSH abundance are characteristic of colitis [57, 68], mainly due to the enrichment of opportunistic pathogens induced by CA administration [37]. The present study further found that a grain-based diet significantly triggered an inflammatory response, mainly by promoting the formation of MBL involved in the lectin pathway of the colonic mucosa. The lectin pathway is initiated by the binding of MBL or ficolins to mannose-containing glycoproteins or carbohydrates on pathogenic surfaces [69]. Therefore, our present findings provide important clues regarding the relationship between BA-metabolizing MAGs and the intestinal health of dairy cows.

Collectively, our genome-centric analysis of the BA transformation pathways of 108 intestinal content samples from dairy cows identified 372 high-quality MAGs that were involved in BA deconjugation, oxidation, and dehydroxylation pathways. Compared with the human and pig gut microbiomes, that of dairy cows had a lower proportion and diversity of microorganisms involved in BA transformation, characterized by the enrichment of host-derived conjugated BAs in the duodenum and jejunum and the prevalence of microbiota-derived unconjugated BAs in the large intestine. Comparative genome analysis showed that *BSH*-carrying microbial populations withstood stressful intestinal conditions via the ability to degrade host mucin, sporulation, and thiamine biosynthesis. The classification of 439 BSH homologs from the intestinal microbiome of dairy cows into 12 clusters based on SSN revealed diverse evolution, taxonomy, signal peptides, and ecological niches for the different clusters. Further integration of microbial genomic signatures, targeted BA pools, and host transcriptomes showed that the strains of *Firmicutes bacterium CAG-110* processed the increased abundance of BSHs from Cluster 1, coinciding with changes in the colon concentration of CA, a compound intricately linked to intestinal inflammation via the lectin pathway after grain intervention. Therefore, our research lays the foundation for a better understanding of microbial BA transformation pathways in the intestine of dairy cows, providing insight into the manipulation of intestinal microbiota to improve the host health.

## Supplementary information

Supplementary figure and table legends

Fig. S1

Fig. S2

Fig. S3

Fig. S4

Fig. S5

Fig. S6

Table S1

Table S2

Table S3

Table S4

Table S5

Table S6

Table S7

Table S8

Table S9

Table S10

Table S11

Table S12

Table S13

## Data Availability

Raw sequence reads for all samples are available under European Nucleotide Archive (ENA) project PRJNA723218. All MAGs produced and utilized in this study have been deposited in Figshare (10.6084/m9.figshare.14456637 and 14176574). Representative MAGs for the human gut with a high-quality genome (*N* = 2935) were downloaded from https://github.com/snayfach/IGGdb. Representative MAGs for the pig gut with a high-quality genome (*N* = 2564) were downloaded from https://db.cngb.org/search/project/CNP0000824/.
