# Supplementary material for: Genome-centric investigation of bile acid metabolizing microbiota of dairy cows and associated diet-induced functional implications
Source: ISME J. 2022 Oct 19;17(1):172–84. doi: 10.1038/s41396-022-01333-5 (PMC9750977; doi:10.1038/s41396-022-01333-5)
Supplement: Supplementary file 1 — Supplementary figure and table legends [file 41396_2022_1333_MOESM1_ESM.docx]

**Supplementary figure and table legends**

**Fig. S1** Bile acid transformation pathways constructed for 372 metagenome-assembled genomes (MAGs) of intestinal microorganisms from dairy cows. **A** Deconjugation of GCA /TCA, and subsequent conversion to 7-keto-CA by *7α-HSDH* or CA-CoA by *baiB*. **B** Deconjugation of GCDCA/TCDCA and subsequent conversion to 7-dehydro-CDCA. **C** Conversion of CDCA to CDCA-CoA via the bai pathway. **D** Conversion of UDCA to UDCA-CoA via the bai pathway. GCA, glycocholic acid; TCA, taurocholic acid; CA, cholic acid; CDCA, chenodeoxycholic acid; GCDCA, glycochenodeoxycholate; TCDCA, taurochenodeoxycholate; UDCA, ursodeoxycholic acid.

**Fig. S2** Comparison of milk quality between the forage-based (F) and grain-based (G) diets. Significance based on the relative index of each cohort according to a *t*-test. **p* < 0.05, ***p* < 0.01, ****p* < 0.001.

**Fig. S3** Comparison of the fermentation parameters between the forage-based (F) and grain-based (G) diets in the rumen (**A**) and colon (**B**). Significance based on the relative index of each cohort according to a *t*-test. **p* < 0.05, ***p* < 0.01, ****p* < 0.001.

**Fig. S4** Principal coordinate analysis (PCoA) plot of (**A**) 978 high-quality metagenome-assembled genomes (MAGs) and (**B**) 372 bile acid (BA)-metabolizing MAGs between the forage-based (F) and grain-based (G) diets in the colon.

**Fig. S5** Changed abundance of 439 bile salt hydrolase homologs after grain introduction.

**Fig. S6** Differentially expressed genes related to the host inflammatory response in the colonic mucosa of dairy cows between the forage-based (F) and grain-based (G) diets. The values are presented as Log_2_FC.

**Table S1.** Ingredients and nutritional compositions of the forage-based (F) and grain-based (G) diets.

**Table S2.** Primer sequences of genes related to the host inflammatory response in the colonic mucosa used for qRT-PCR.

**Table S3.** Genomic statistics for 978 high-quality metagenome-assembled genomes (MAGs) from 108 content samples covering six intestinal regions in 18 dairy cows.

**Table S4.** Proportions of the GTDB classification for 978 high-quality metagenome-assembled genomes (MAGs).

**Table S5.** Genes involved in bile acid (BA) transformation pathways from 372 BA-metabolizing metagenome-assembled genomes (MAGs).

**Table S6.** Bile acid metabolism in 2 935 human metagenome-assembled genomes (MAGs, completeness ≥ 90% and contamination < 5%) and 2 564 pig MAGs (completeness > 80% and contamination < 5%).

**Table S7.** Bile acid profiles in the duodenum, jejunum, ileum, cecum, colon, and rectum of dairy cows.

**Table S8.** CAZyme-predicted proteins of 368 *BSH*-carrying metagenome-assembled genomes (MAGs) in six intestinal regions of dairy cows.

**Table S9.** KEGG orthologous groups of 368 *BSH*-carrying metagenome-assembled genomes (MAGs) in six intestinal regions of dairy cows.

**Table S10.** Genome properties of 368 *BSH*-carrying metagenome-assembled genomes (MAGs) in six intestinal regions of dairy cows.

**Table S11.** Protein sequence similarity network (SSN) of bile salt hydrolases (BSHs).

**Table S12.** Prediction of signal peptides on 439 bile salt hydrolase homologs by SignalP 6.0.

**Table S13.** Significantly different abundance of 46 bile salt hydrolases between the forage-based (F) and grain-based (G) diets in the colons of 12 dairy cows.
