# Supplementary figures and images for: Genome-centric investigation of bile acid metabolizing microbiota of dairy cows and associated diet-induced functional implications
Source: ISME J. 2022 Oct 19;17(1):172–84. doi: 10.1038/s41396-022-01333-5 (PMC9750977; doi:10.1038/s41396-022-01333-5)

**A**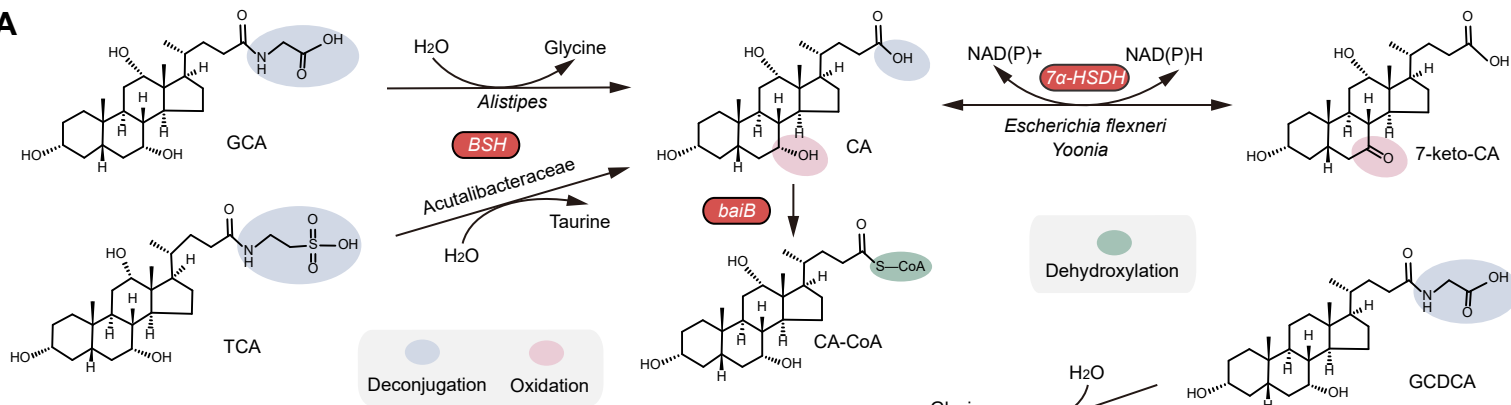**B**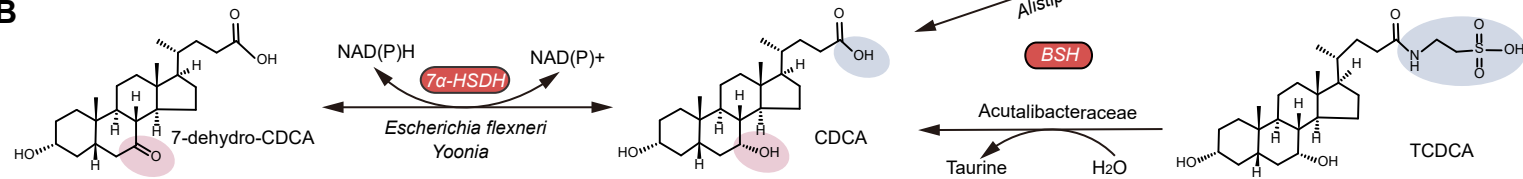**C**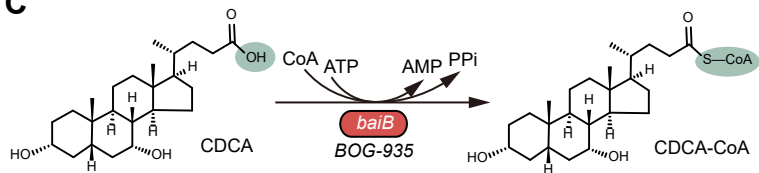**D**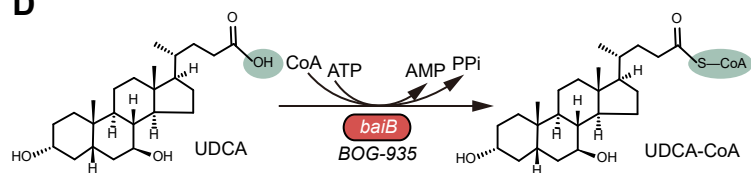

Supplement: Supplementary file 2 — Fig. S1 [file 41396_2022_1333_MOESM2_ESM.pdf]

Milk fat (%)

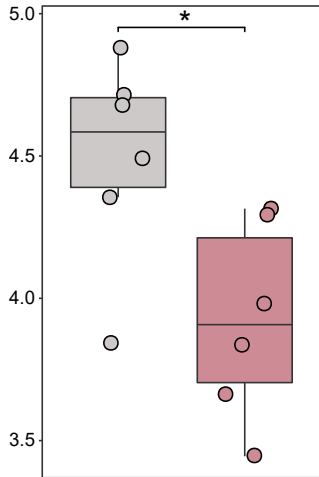

Milk protein (%)

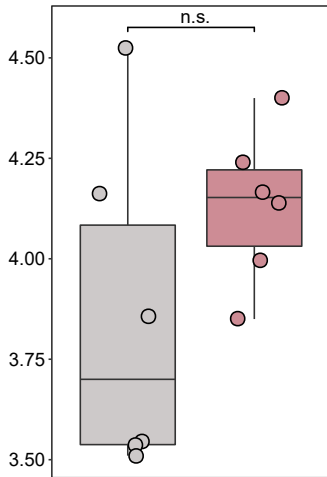

Lactose (%)

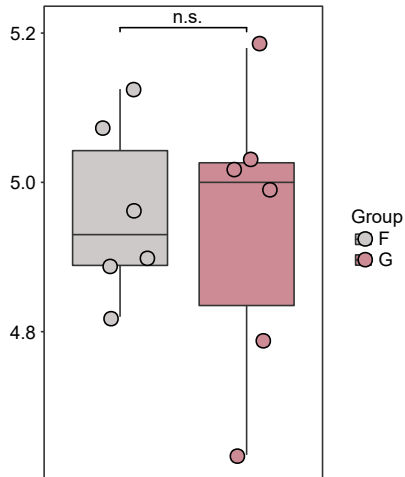

Supplement: Supplementary file 3 — Fig. S2 [file 41396_2022_1333_MOESM3_ESM.pdf]

**A**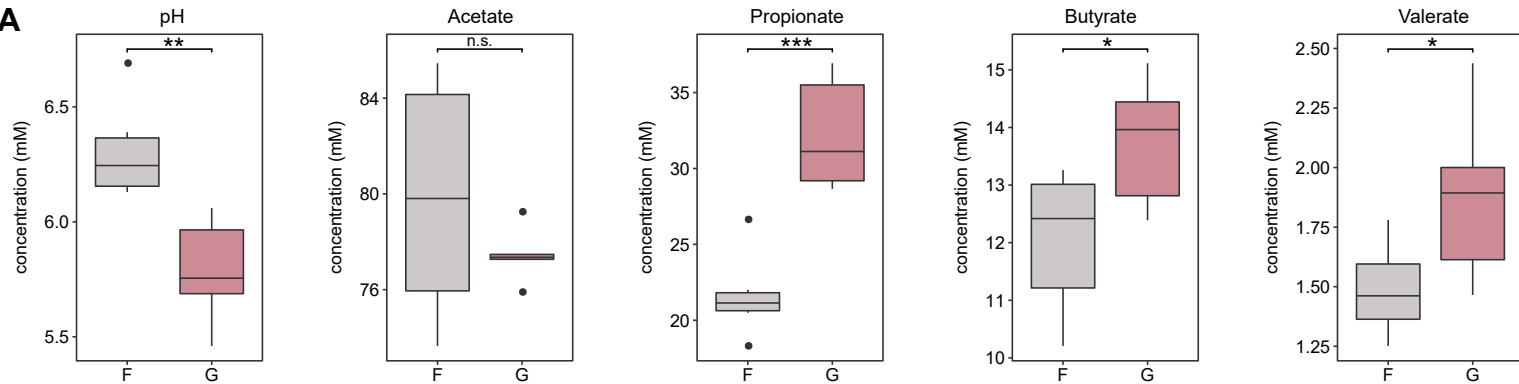**B**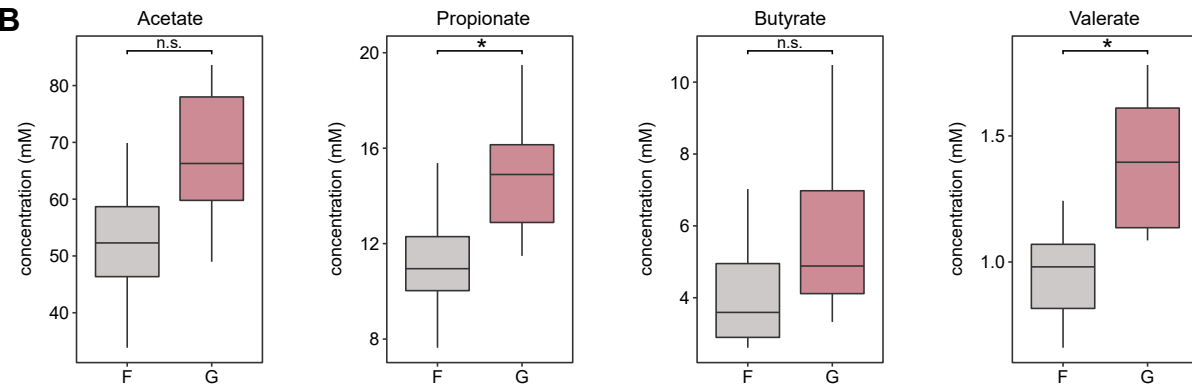

Supplement: Supplementary file 4 — Fig. S3 [file 41396_2022_1333_MOESM4_ESM.pdf]

**A**ANOSIM:  $R = 0.209$   $p = 0.002$ 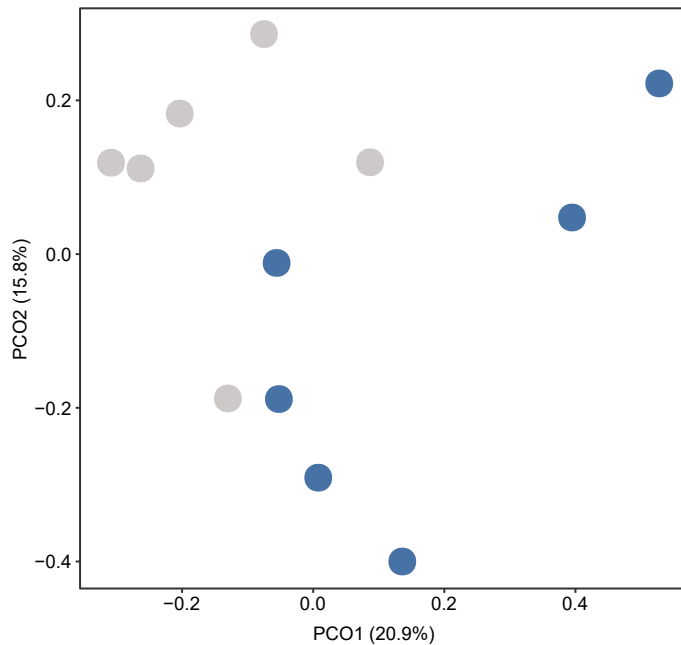**B**ANOSIM:  $R = 0.261$   $p = 0.002$ 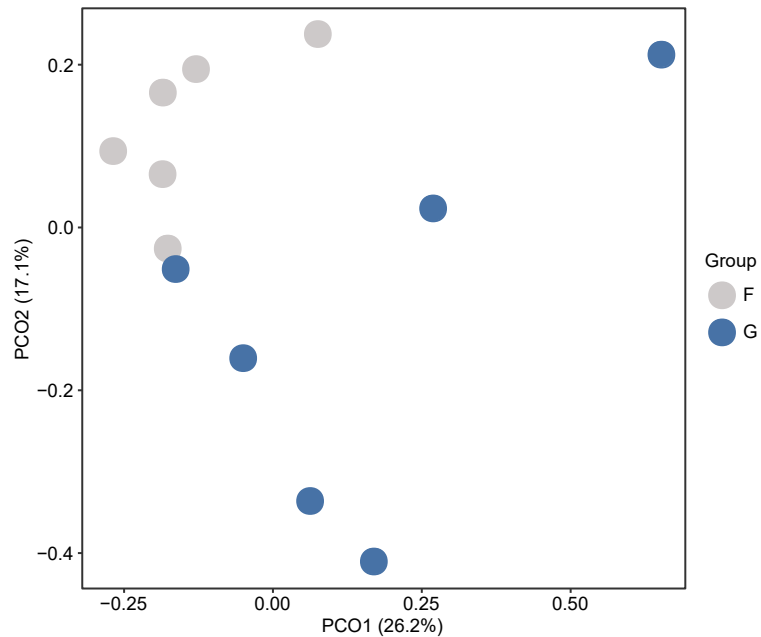

Supplement: Supplementary file 5 — Fig. S4 [file 41396_2022_1333_MOESM5_ESM.pdf]

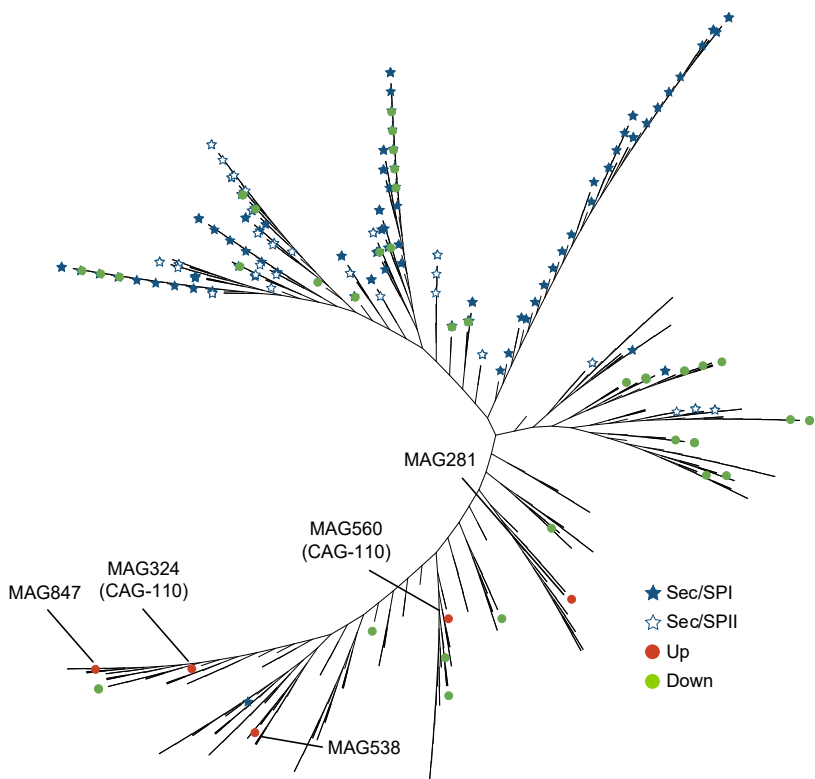

Supplement: Supplementary file 6 — Fig. S5 [file 41396_2022_1333_MOESM6_ESM.pdf]

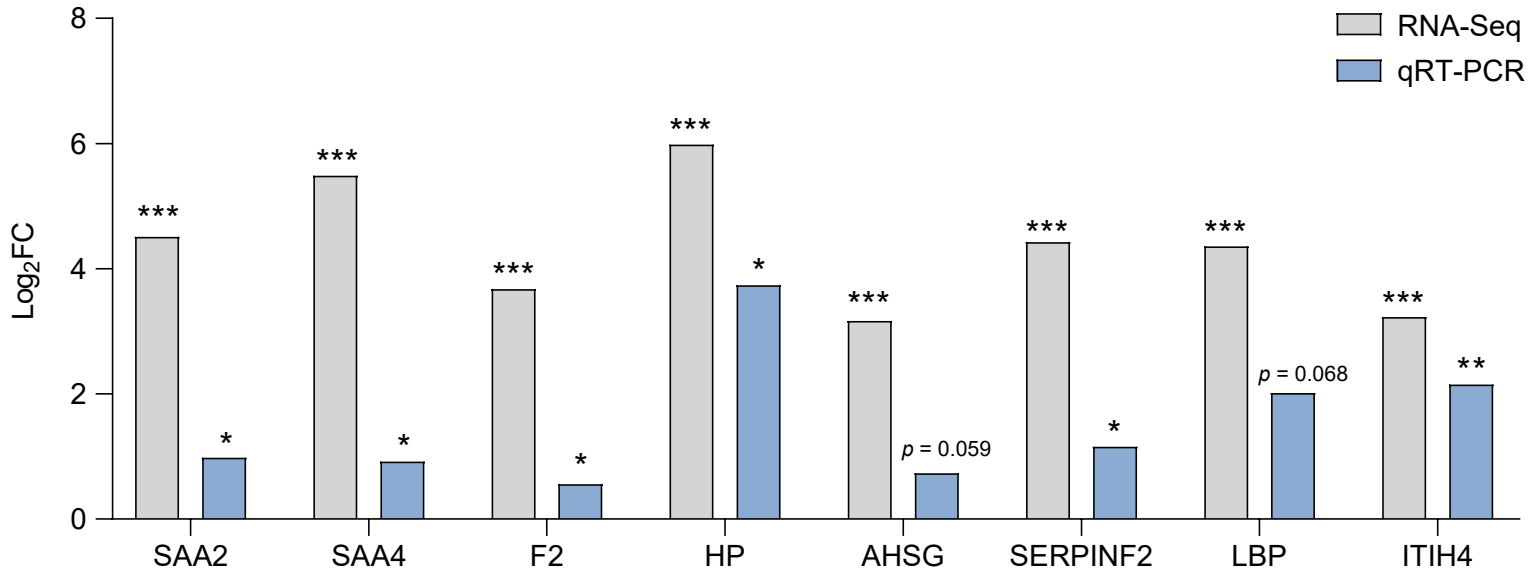

Supplement: Supplementary file 7 — Fig. S6 [file 41396_2022_1333_MOESM7_ESM.pdf]
