# Supplementary material for: Genome-centric investigation of bile acid metabolizing microbiota of dairy cows and associated diet-induced functional implications
Source: ISME J. 2022 Oct 19;17(1):172–84. doi: 10.1038/s41396-022-01333-5 (PMC9750977; doi:10.1038/s41396-022-01333-5)
Supplement: Supplementary file 9 — Table S2 [file 41396_2022_1333_MOESM9_ESM.docx]

**T****able S2.** Primer sequences of genes related to the host inflammatory response in the colonic mucosa for qRT-PCR.

| Gene name | Accession number or source | Primer sequence (5’→3’) | Amplicon size (bp) |
| --- | --- | --- | --- |
| Bos taurus inter-alpha-trypsin inhibitor heavy chain 4 (ITIH4) | NM_001015590.3 | For: TGGGCATACCTGACCATCCA | 248 |
|  |  | R: CCCACAGAATGACCAAACCTCA |  |
| Bos taurus lipopolysaccharide binding protein (LBP) | NM_001038674.2 | For: TCAAGCAACAGCCCAAATGC | 161 |
|  |  | R: TGGCGGTGTTGAAGGCATAA |  |
| Bos taurus haptoglobin (HP) | NM_001040470.2 | For: TTCGCTATCAGTGCAAACCCT | 128 |
|  |  | R: GCACACTGCCTCACATTCAG |  |
| Bos taurus serum amyloid A4, constitutive (SAA4) | NM_001040505.2 | For: TTCTGGAGGGACGAGGCTAT | 105 |
|  |  | R: CACCTCTGGGTACCTGTCTC |  |
| Bos taurus serum amyloid A2 (SAA2) | NM_001075260.2 | For: TTTAAGGGTACGACCAGTGGC | 126 |
|  |  | R: TCAGTACTTGTCAGGCAGGC |  |
| Bos taurus coagulation factor II, thrombin (F2) | NM_173877.1 | For: ACCGCTGGTATCAAATGGGC | 152 |
|  |  | R: CCTTGGAATGTGGGTGGCTC |  |
| Bos taurus alpha 2-HS glycoprotein (AHSG) | NM_173984.3 | For: AAGCGTGGTAGCAGTTCCC | 103 |
|  |  | R: GAACGCTTCTCCCGAGGATG |  |
| Bos taurus serpin family F member 2 (SERPINF2) | NM_174670.2 | For: ACGGAAGGGAAGATCGAGGA | 122 |
|  |  | R: CTCTGTGTGAGGTTCGGGTC |  |
| GAPDH | NM_001034034.2 | For: AGATGGTGAAGGTCGGAGTGA | 192 |
|  |  | R: TCCCGTTCTCTGCCTTGACT |  |
