# Supplementary material for: Genome-centric investigation of bile acid metabolizing microbiota of dairy cows and associated diet-induced functional implications
Source: ISME J. 2022 Oct 19;17(1):172–84. doi: 10.1038/s41396-022-01333-5 (PMC9750977; doi:10.1038/s41396-022-01333-5)
Supplement: Supplementary file 14 — Table S7 [file 41396_2022_1333_MOESM14_ESM.docx]

**Table S7.** Bile acid profiles in the duodenum, jejunum, ileum, cecum, colon, and rectum of dairy cows

| **Metabolite** | **Duodenum** | **Jejunum** | **Ileum** | **Cecum** | **Colon** | **Rectum** |
| --- | --- | --- | --- | --- | --- | --- |
| TCA | 56833.87 ± 11974.623 | 92031.67 ± 13137.322 | 260.52 ± 53.081 | 26.61 ± 5.155 | 18.24 ± 3.168 | 25.69 ± 8.054 |
| TCDCA | 1931.92 ± 417.555 | 3580.83 ± 605.162 | 20.62 ± 2.173 | 7.1 ± 0.904 | 4.68 ± 0.903 | 6 ± 0.903 |
| CA | 6241.17 ± 2716.402 | 38685.38 ± 6366.901 | 1021.8 ± 62.552 | 69.99 ± 13.485 | 26.68 ± 11.308 | 85.3 ± 25.45 |
| GCA | 36917.99 ± 6102.772 | 40968.63 ± 9375.477 | 182.93 ± 23.122 | 31.7 ± 13.247 | 38.62 ± 15.999 | 35.82 ± 15.259 |
| GCDCA | 1484.13 ± 265.831 | 2058.59 ± 468.554 | 24.47 ± 1.234 | 6.38 ± 1.405 | 6.82 ± 1.331 | 5.98 ± 1.302 |
| CDCA | 153.93 ± 77.935 | 851.7 ± 192.315 | 81.15 ± 2.193 | 10.01 ± 0.783 | 8.07 ± 0.579 | 12.5 ± 1.813 |
| TDCA | 8923.06 ± 2101.358 | 14522.01 ± 2535.486 | 51.64 ± 7.304 | 19.74 ± 2.668 | 13.88 ± 2.147 | 18.73 ± 3.974 |
| TLCA | 120.74 ± 19.268 | 182.62 ± 26.99 | 5.82 ± 0.535 | 2.92 ± 0.342 | 2.1 ± 0.291 | 2.22 ± 0.249 |
| UCA | 34.74 ± 1.66 | 37.9 ± 2.038 | 0.55 ± 0.058 | 2.78 ± 0.171 | 0.84 ± 0.058 | 3.02 ± 0.098 |
| αMCA | 0.09 ± 0.02 | 0.67 ± 0.106 | 0.2 ± 0.044 | 0.45 ± 0.123 | 0.54 ± 0.032 | 0.58 ± 0.039 |
| βMCA | 0.44 ± 0.182 | 6.26 ± 0.978 | 1.22 ± 0.062 | 0.43 ± 0.053 | 0.34 ± 0.019 | 0.5 ± 0.057 |
| GDCA | 4773.83 ± 1041.422 | 5579.47 ± 1005.195 | 43.86 ± 2.586 | 15.23 ± 4.06 | 15.34 ± 4.73 | 16.66 ± 6.211 |
| GLCA | 58.58 ± 5.631 | 51.84 ± 5.32 | 1.04 ± 0.176 | 1.29 ± 0.096 | 0.45 ± 0.062 | 1.45 ± 0.088 |
| UDCA | 0.14 ± 0.038 | 2 ± 0.384 | 1.17 ± 0.077 | 1.61 ± 0.311 | 1.5 ± 0.111 | 1.24 ± 0.317 |
| NorDCA | 1.51 ± 0.208 | 5.26 ± 1.02 | 0.72 ± 0.092 | 2.53 ± 0.23 | 1.3 ± 0.116 | 2.4 ± 0.16 |
| DCA | 790.69 ± 430.054 | 4253.34 ± 772.987 | 369.75 ± 21.828 | 1435.84 ± 46.231 | 923.14 ± 80.975 | 1328.1 ± 81.146 |
| βDCA | 1.72 ± 0.785 | 6.33 ± 1.318 | 6.7 ± 0.18 | 196.84 ± 16.374 | 64.07 ± 5.324 | 165.85 ± 12.762 |
| LCA | 10.92 ± 1.649 | 16.29 ± 1.498 | 11.63 ± 0.374 | 152.75 ± 8.821 | 118.9 ± 9.369 | 145.65 ± 10.022 |
| isoalloLCA | 0.08 ± 0.019 | 0.28 ± 0.044 | 1.15 ± 0.659 | 3.88 ± 0.451 | 4.19 ± 0.507 | 5.28 ± 0.512 |
| isoLCA | 0.21 ± 0.089 | 0.47 ± 0.11 | 1.09 ± 0.416 | 13.08 ± 0.949 | 11.19 ± 0.948 | 19.84 ± 1.433 |
| 12-KetoLCA | 48.37 ± 4.059 | 68.81 ± 4.584 | 11.42 ± 0.403 | 150.05 ± 15.528 | 461.57 ± 35.284 | 296.1 ± 21.015 |
| dehydroLCA | 33.89 ± 1.631 | 30.57 ± 1.375 | 2.54 ± 0.488 | 9.18 ± 0.626 | 45.47 ± 5.673 | 24.64 ± 2.708 |
| 6-KetoLCA | 0.21 ± 0.051 | 0.89 ± 0.637 | 0.84 ± 0.038 | 1.02 ± 0.144 | 0.7 ± 0.049 | 0.53 ± 0.175 |
| 7-KetoLCA | 0.99 ± 0.288 | 38.98 ± 10.931 | 23.52 ± 1.121 | 2.62 ± 0.166 | 1.37 ± 0.154 | 2.37 ± 0.502 |
| 7-DHCA | 381.25 ± 88.333 | 1769.85 ± 189.196 | 283.98 ± 28.354 | 12.5 ± 1.861 | 4.4 ± 0.333 | 15.04 ± 3.079 |
| 12-DHCA | 13.34 ± 1.944 | 54.73 ± 5.33 | 10 ± 0.45 | 2.17 ± 0.232 | 1.09 ± 0.082 | 3.75 ± 0.686 |
| 3-DHCA | 13.82 ± 3.223 | 64.78 ± 9.505 | 6.03 ± 0.275 | 1.27 ± 0.139 | 0.68 ± 0.067 | 3.06 ± 0.767 |

Values are mean ± SEM concentrations (nmol/g intestinal contents) measured using UPLC-MS/MS.
